# Tze-Yun Leong: the need for intelligent regulation

**DOI:** 10.2471/BLT.20.030420

**Published:** 2020-04-01

## Abstract

Tze-Yun Leong talks to Gary Humphreys about the challenges faced in realizing the potential of digital health.

Q: You started out in electrical engineering. How did you get from there to working on artificial intelligence (AI)?

A: It started with a fascination for computers. I still remember my first computer class. The teacher came to class dressed like a magician, with a pointed hat and a purple cape, and he said, ‘computing is like magic’. I believed it then and still do. I studied and graduated with degrees in computer science and electrical engineering, so I was trained in working with software and hardware as a whole system and in real-world applications. I think this system perspective has helped shape my research interests and directions throughout my career.

Q: What was your first contact with artificial intelligence?

A: I worked on a research project called “Murmur Clinic: An Auscultation Expert System” – the objective was to build a computer program that would diagnose the symptoms and sound patterns related to different heart conditions. It was really exciting to see the system predictions actually matching the diagnoses of the doctors!

Q: What is the main focus of your research?

A: Developing human-aware AI that is capable of dealing with real-life problems. Essentially, designing AI innovations that work for, with and alongside humans. To do this, the AI needs to be able to make good decisions despite incomplete information, evolving conditions and limited resources.

Q: Can you give some examples of AI applications you have worked on?

A: Specific applications include effective management of coronary artery disease patients with stroke, follow-up strategies for colorectal cancer patients after surgery, interpretation of intensive care unit (ICU) data for decision support, etc. A lot of my work has been on neurocognitive issues, with applications ranging from intelligent image-based diagnosis in strokes and brain injuries, diagnosis and staging for dementia, and personalized assistive care for older people with neurodegenerative disorders. Currently, I am also working on future research directions of biomedical and health informatics, and ethics and governance, and regulatory considerations for AI in health in general.

Q: What are the main AI-related ethics, governance, and regulatory concerns?

A: They cover a broad range of issues reflecting the extraordinarily broad application of AI in the contemporary health space. Just to focus on clinical application, AI supports diagnosis, treatment, and prognosis management through data analytics and predictive modelling for disease screening, staging, progression and risk stratification, individualized treatment and care plans, optimization of workflow processes and cost-effectiveness analyses. AI is also essential in scientific discovery and experimentation, especially in drug discovery and clinical trials, as well as in disease modelling and surveillance, and health policy analysis and design.

In all of these applications, digital health and AI brings tremendous benefits, but at the same time sometimes unexpected, and even harmful effects. As we learn more about the power and the limitations of digital health and AI in medicine, we need to catch up with improving the development and application approaches and processes. We also need to start establishing relevant regulatory and governance guidelines and protocols to enhance the benefits and minimize the potential harm of these technologies and systems.

The main danger and the greatest harms are brought about by poorly designed, hastily developed, inadequately tested, and/or inappropriately used digital and AI health systems. Unfortunately, we have heard many horror stories, which result from such situations. So much needs to be done to improve the design, development, implementation, use, and regulation of digital and AI health system applications.

“We [need] relevant regulatory and governance guidelines and protocols to enhance the benefits and minimize the potential harm of these technologies and systems.”

Q: What can be done to address concerns about digital data privacy?

A: Drawing up and enforcing robust privacy regulation, such as the European Union General Data Protection Regulation and the Singapore Personal Data Protection Act is a good place to start. Privacy-preserving technologies like encryption, de-identification, and anonymization also have an important role to play. Recent developments in this area include homomorphic encryption, which allows users to work with data while they are still encrypted, differential privacy, which permits the sharing of information about a dataset while withholding information about individuals in the dataset, and federated machine learning, where data are processed in separate locations. However, such innovations are only part of the answer. Many of the privacy breaches in health care result from administrators or clinicians leaving files with private information open after use, accessing data without authorization, or inadvertently uploading unprotected files or personal information online. Ensuring digital literacy and cyber-awareness among health professionals is therefore key.

Q: Health systems seem to be increasingly targeted by cyber-criminals. What can be done to address this problem?

A: On the cyber-security front, we need to make it as hard as possible for hackers to get into systems and steal data or introduce ransomware and/or viruses. This will require tightening up both physical and digital security, and ensuring that the operational and workflow processes are well designed and protected. The reality is that no system is 100% secure. AI itself can play an important role with intelligent monitoring and prediction to identify suspicious activities.

Q: To what extent should we be concerned about data-derived bias in the development and application of AI?

A: It is important to remember that AI does not have ethical values or biases. AI systems execute computational processes that take in a set of data or other forms of information, and generate an output or answer based on some pre-defined steps structured as algorithms, or recipes. The data we feed into the AI systems reflect the biases and problems inherent in the approaches and processes used to generate that data in the first place.

“AI is not intended to replace clinicians.”

Q: There is increasing concern about the impenetrability of so-called black box algorithms. What can be done to ensure transparency in algorithm processes and outputs?

A: A system that is conceived, designed, built and tested with proper oversight can be trusted to work according to its original intention. If I can make an analogy: when we board an airplane we trust that the pilot will expertly and safely fly us to our destination, but we don’t expect the pilot to understand the math that went into the design of the turbine blades or the chemistry that went into the composites used in the airframe.

Q: But if a doctor wants to know how the algorithm is making a certain decision regarding a care plan for a specific patient shouldn’t he or she be able to?

A: Well-designed AI for health should have clear documentation of the decision technologies used and the data relied on, as well as usage guidelines and instructions on how to verify the recommendations made and how to check for problems. All this information should be presented at different levels so that the target users, such as doctors, patients and maintenance technicians, can understand and work with it. Such systems can be “transparent”, without the need for every computational step to be traceable. That said, it is important to recognize that this is a sensitive area. Currently, it is impossible for the AI to make a full assessment of the patient in the way that a clinician can, accounting for the particularities of each patient, including their nutritional and socioeconomic status etc., so it is essential that clinicians make final determinations, using AI as an expert assistant rather than a surrogate. A core goal of AI in medicine is to help clinicians, especially those junior clinicians with less experience, to make more accurate diagnoses to support more effective treatment and better outcomes. The key words here are ‘help’ and ‘support’. AI is not intended to replace clinicians, and we may never want fully “trusted” AI in health systems that would make autonomous decisions.

Q: What do you consider the main challenges in regard to AI in the clinical space?

A: The biggest challenge is for AI to provide consistent, effective support for all the major clinical decision-making tasks in different contexts. Beyond that, AI in health risks undermining the clinician’s autonomy, just as digital navigation apps and calculators undermine our abilities to navigate and count. It is therefore important to identify the skills that should be preserved, enhanced, or augmented for clinicians of the future. On the upside, AI and digital health systems can already help clinicians navigate the vast and rapidly advancing biomedical knowledge space to source relevant, information, and conduct virtual scenario analyses.

Q: Given the complexity of the digital space, how confident are you that regulators have sufficient capacity to meet their responsibilities?

A: Ministries and regulators in many countries are engaging health informaticians, computer scientists familiar with health applications, or health care professionals well versed in information and computing technology, in the working groups and task forces to define the relevant rules and guidelines. However, digital and AI technologies are advancing very quickly, and there is no doubt that regulators are faced with a big challenge. But they are not alone.

As we move into the fourth industrial revolution, the education systems have to catch up, providing the appropriate education and training for everyone –scientists, engineers, researchers, developers, managers, and users of digital health systems, including, of course, health professionals and regulators. Specifically, we should recognize that computational problem solving is the fourth basic skill that everyone needs – in addition to the 3Rs of reading, writing, and arithmetic. Achieving digital literacy would help us understand the strengths and limitations of the different digital systems and realize their full potential.

